# Supplementary material for: Multidimensional diffusion MRI reveals heterogeneous microstructural remodeling associated with amyloid pathology
Source: bioRxiv. 2026 Sep 1:2026.08.26.747377. Preprint. [Version 1] doi: 10.64898/2026.08.26.747377 (PMC13684513; doi:10.64898/2026.08.26.747377)
Supplement: Supplementary file 1 [file media-1.pdf]

Sample	Weight (g)	Sex	Age at perfusion (days)	Time between perfusion and scan (days)
WT1	24	F	258	270
WT2	24	F	258	266
WT3	24	F	258	333
WT4	25	F	258	275
AD1	21	F	234	340
AD2	24	F	234	228
AD3	22	F	234	341
AD4	21	F	234	235

Supp Table 1. details of subjects, dates of perfusion and dates of chosen scans

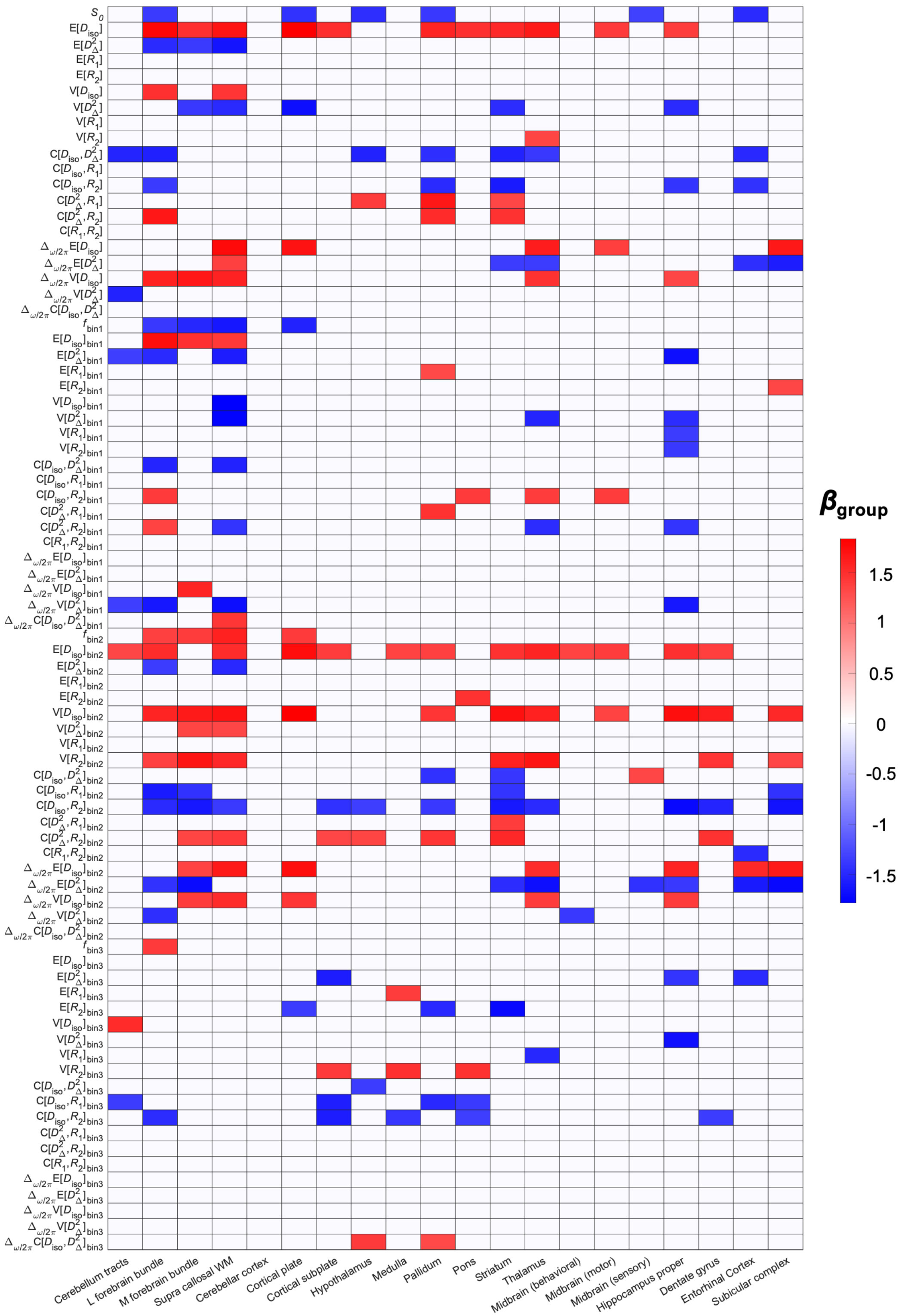

Supp Fig 1. Heatmap of linear regression effect sizes β_{group} with all metrics. Only entries where the difference is significant (p<0.05) are colored for viewability. Along the x-axis are the metrics, in the order of S_0, $\mathrm{E}[x]$, $\mathrm{V}[x]$, $\mathrm{C}[x, y]$, $\Delta_{\omega/2\pi}$ metrics, and then corresponding bin 1-, 2- and 3-resolved metrics. Along the y-axis are the ROIs.
